# Statistical model comparison applied to common network motifs

**DOI:** 10.1186/1752-0509-4-18

**Published:** 2010-03-03

**Authors:** Núria Domedel-Puig, Iosifina Pournara, Lorenz Wernisch

**Affiliations:** 1 Departament de Física i Enginyeria Nuclear, Universitat Politècnica de Catalunya, Edifici GAIA, Rambla de Sant Nebridi s/n 08222, Terrassa, Barcelona, Spain; 2 School of Crystallography, Birkbeck College, University of London, Malet Street, London WC1E 7HX, UK; 3 MRC Biostatistics Unit, Robinson Way, Cambridge CB2 0SR, UK

## Abstract

**Background:**

Network motifs are small modules that show interesting functional and dynamic properties, and are believed to be the building blocks of complex cellular processes. However, the mechanistic details of such modules are often unknown: there is uncertainty about the motif architecture as well as the functional form and parameter values when converted to ordinary differential equations (ODEs). This translates into a number of candidate models being compatible with the system under study. A variety of statistical methods exist for ranking models including maximum likelihood-based and Bayesian methods. Our objective is to show how such methods can be applied in a typical systems biology setting.

**Results:**

We focus on four commonly occurring network motif structures and show that it is possible to differentiate between them using simulated data and any of the model comparison methods tested. We expand one of the motifs, the feed forward (FF) motif, for several possible parameterizations and apply model selection on simulated data. We then use experimental data on three biosynthetic pathways in *Escherichia coli *to formally assess how current knowledge matches the time series available. Our analysis confirms two of them as FF motifs. Only an expanded set of FF motif parameterisations using time delays is able to fit the third pathway, indicating that the true mechanism might be more complex in this case.

**Conclusions:**

Maximum likelihood as well as Bayesian model comparison methods are suitable for selecting a plausible motif model among a set of candidate models. Our work shows that it is practical to apply model comparison to test ideas about underlying mechanisms of biological pathways in a formal and quantitative way.

## Background

Cellular processes are very complex, but it seems that such processes can often be broken down into a small number of reoccuring patterns of interconnections known as *network motifs *[1,2]. Interestingly, some motifs are known to display specific dynamic functional roles [3,4]. Motif dynamics can now be assessed in a precise manner thanks to the emergence of new experimental techniques that allow generating high quality time series data with a high temporal sampling rate [5-9]. However, studying biological systems in general involves two steps: first, the *components *of the network need to be identified, and then the type of *relationships *between them established. Different methods exist for doing so. While some have focused on deriving pairs of possible interacting molecules from existing databases [1], others have tried to reconstruct networks from scratch integrating different sources of both static and dynamic data [10]. In fact, automatic identification of interactions has been the aim of reverse engineering for many years [11]. In general, multiple hypotheses about the architecture of a network are easily generated, and assessing their validity is often difficult.

Existing knowledge can be used in the model identification process. If certain aspects of the networks are already known, the space of possible models is smaller and reverse engineering amounts to *model comparison *in this case. Several techniques exist for comparing the plausibility of different candidate models, given observations. On the one hand, judgement is sometimes based solely on visual inspection of predicted *vs. *observed data [12]. On the other hand, formal frequentist methods such as likelihood ratio tests (LRT) and bootstrapping have been used for comparing models [13]. Until recently, Bayesian model comparison approaches such as Bayes factors have largely been ignored in analysing the identifiability of biological systems from time series data [14]. However, Bayesian approaches to problem solving have recently gained in popularity due to their inherent control of complexity and the ease with which any prior knowledge about the system under study can be incorported (for an introduction to Bayesian modelling see [15]). This prior information is derived either through literature surveys or through experimental observations.

In this paper, we wish to show how formal statistical model comparison methods can be applied to a typical systems biology scenario. We analyze whether the dynamic fingerprint of a number of commonly occurring motifs allows discrimination of the underlying network structure based on simulated data. These are the single input motif (SIM), regulatory chain (RC), feedforward (FF) and feedback (FB) motifs. Any of these motif architectures can be parameterized differently when converted to ordinary differential equations resulting in different dynamics of the system. This has been reported in the case of the FF motif [5-7], which appears in the arabinose, flagella, and galactose pathways in *E. coli*. The original work on these systems explored the differences between each FF type and a control non-FF architecture in a statistically informal manner [5-7]. Here we take the analysis one step further and explore whether statistical model comparison is also able to distinguish between FF parameterizations.

Before we apply model comparison to specific dynamic models derived from biological network motifs (Section Results), we provide some background on statistical model comparison in the next section (Section Methods). The spirit of this work is to demonstrate in a didactic way how different statistical model comparison tools perform on a class of dynamic models of interest in the systems biology community. We have therefore restricted the type and number of methods and models to those most easily implementable and accessible to the non-expert reader. In addition, we provide an introductory exercise with a simple one-equation model in [Additional file 1]. The statistical methods presented have important limitations, which we also briefly discuss.

## Methods

A model *M *describes the deterministic dynamic behaviour of variables *z*(*t*) = (*z*_1_(*t*), ..., *z*_*K *_(*t*))^*T *^by a system of differential equations *dz*_*k*_/*dt *= *f*_*k*_(*z*(*t*), *t*, *θ*), using parameters *θ*. The parameters may include initial values. Measurements *y*_*ki *_ of each *z*_*k*_ are taken at time points *t*_*i*_, *i *= 1, ..., *N*. For notational simplicity, we assume all variables are measured at all the time points, but the following discussion is easily extended to more general cases. An additive Gaussian measurement error ϵ ~ *N *(0, *σ*^2^) affects the observations of the variables. For simplicity we assume here that the error distributions of the variables are independent and constant over time. They are also characterised by some variance *σ*^2 ^which we consider to be part of the parameters.

The probability of the data *Y *given some estimates  of the parameters is(1)

where *p*_*N *_(*y *| , *σ*^2^) is a Gaussian with mean  and variance *σ*^2^. Estimates _*ki *_ are obtained by  where  are the solutions of differential equation system *z*_*k*_(*t*) = *f*_*k*_(*z*(*t*), *t*, ).

Model comparison methods involve two steps: first the model parameters need to be inferred from the available data, and then the adequacy of each calibrated model needs to be assessed. The classical and Bayesian approaches to each step are described next.

### Bayesian inference

In Bayesian statistics our knowledge about model parameters, conditional on the observed data, is summarised by probability distributions. This is allowed because parameters are random variables with a degree of uncertainty. The relationship between the data and the parameters is described by(2)

The posterior distribution of the parameters *θ*, given the data *Y*, is proportional to the likelihood *P *(*Y *| *θ*, *M*) of the parameters times the parameter prior *P *(*θ *| *M*), normalised by the likelihood or evidence *P *(*Y *| *M*) for model *M*. Samples from the posterior distributions of model parameters are routinely obtained by Markov Chain Monte Carlo (MCMC) methods [16]. Descriptions of the posterior distribution in terms of mean, median or variance are easily obtained from such samples. In the following, estimates of the 95% credible interval (CI), for example, are obtained by the cutoffs for the lowest 2.5% and highest 2.5% of samples.

The denominator in Equation 2, the model evidence, is constant during the calibration step for one particular model and thus can be ignored. However, it becomes our quantity of interest in model comparison. Computation of the model evidence requires solving the integral *p*(*Y *| *M*) = ∫*p*(*Y *| *θ*, *M*) *p*(*θ *| *M*)*dθ*, which is analytically intractable for most examples discussed in this paper. However, if a sample of *S *sample points *θ*_1_, ..., *θ*_*S*_, from the posterior distribution *p*(*θ *| *Y*, *M*) is available (for example, from an MCMC simulation), then the model evidence can be estimated by Gelfand and Dey's [17] reciprocal importance sampler, which is defined as(3)

where *h*(*θ *| *M*) is an arbitrary probability density function over parameters *θ*. The choice of a suitable function *h*(*θ*_*s *_| *M*) is crucial. If set to the prior we obtain the harmonic mean estimator which can perform very poorly due to its high variance. The variance problem is mitigated when a distribution is chosen which is close to the posterior distribution. Stability of the estimator is, for example, achieved by setting *h*(*θ *| *M*) to a multivariate Gaussian fitted to the sampled points *θ*_*s*_, or to a multivariate *t*-distribution as used here. The reciprocal importance sampler was shown to perform well [18] when compared to other model comparison methods such as reversible jump MCMC or simple information criteria like the BIC.

As we show in the simulations below the reciprocal importance sampler is suitable for the comparatively simple models which we investigate in this study. For more complex models, particularly with many modes, more complex model comparison algorithms might be required (see, for example, [14]). Such algorithms are harder to implement and run. The strategy we are suggesting here is to test via simulations whether a particular type of models is amenable to simple model selection procedures based on MCMC samples and only if this is not the case to develop more advanced methods.

### Bayesian model comparison

Given a particular candidate model, *M*_*i*_, its posterior probability is given by(4)

where *p*(*M*_*i*_) is the prior probability of the model, and *p*(*Y *| *M*_*i*_) is the model evidence which we estimate here by equation 3.

According to Occam's principle, simpler models are preferred over complex models if they explain the data equally well. If the unknown parameters *θ *are integrated out as in equation 4, the posterior model probability incorporates a balance between complexity and fit. Since *P *(*Y *| *M*) embodies Occam's principle, it will be the key quantity for model comparison. One way to see that *P *(*Y *| *M*) can be used to choose the model with the better predictive performance is as follows. The model likelihood *P *(*Y *| *M*) essentially captures the sequential predictive power of the model over past incremental data sets, since *P *(*Y *| *M*) = *P *(*Y*_1 _| *M*)*P *(*Y*_2 _| *Y*_1_, *M*) ... *P*(*Y*_*n *_| *Y*_1_, ..., *Y*_*n*-1_, *M*), that is, it captures how well part *Y*_*i *_of the data *Y *= (*Y*_1_, ..., *Y*_*n*_) is predicted using earlier parts *Y*_1_, ..., *Y*_*i*-1 _to calibrate the model. This makes complex models which overfit less likely. An alternative though related measure that penalises models that overfit is the Deviance Information Criterion (DIC) [19], which measures the predictive power on unseen data. It relies more strongly on assumptions about the distribution of future data, but assesses the fully calibrated model, not only versions calibrated by partial data. To define the DIC we first set the deviance as *D*(*y*, *θ*, *M*) = -2 log *p*(*y *| *θ*, *M*). For large sample sizes, the model minimizing the deviance is the model with the highest posterior probability (see, for example, [20]). The predictive deviance for future data can be approximated by(5)

for samples *θ*_*s *_from the posterior (for example, by MCMC simulation) and (*y, M*) = 1/*N *Σ_*s*_*D*(*y*, *θ*_*s*_, *M*) is the average deviance and  = 1/*N *Σ_*s *_*θ*_*s *_is the vector of posterior parameter means. The lower the DIC the better the model. However, we find that the DIC calculations are often unstable resulting in completely unrealistic values, which might be less relied upon.

Note that the complexity of the model is not easily captured by the number of estimated parameters or degrees of freedom, as in the well known Akaike's information criterion (AIC) or even the BIC and related information criteria. If correlated parameters or informative priors are used, for example, the number of *effective *degrees of freedom, pD, is reduced. They can be estimated by (see [19])(6)

Once the probability of the model is known, we can select the most probable model from a set of competitive models using the Bayes factor *BF *= *p*(*Y *| *M*_*i*_)/*p*(*Y *| *M*_*j*_). That is, the Bayes factor measures the extent by which the data increase the odds of *M*_*i *_to *M*_*j*_. Standard cutoffs for interpreting the significance of BFs, like [21], then allow interpretation of the result. Basically, a BF above 1 provides weak, above 3 substantial, above 10 decisive, and above 100 overwhelming evidence for model *M*_*i *_over *M*_*j*_.

The effective degree of freedom measures how many and by how much parameters are constrained by the data. On one hand, each parameter contributes close to one degree if the width of its posterior is small compared to the width of its prior. On the other hand, a parameter contributes very little to the overall effective degrees of freedom if it is not well constrained by the data and the width of its posterior hardly differs from the width of its prior. Consequently, the Bayes factor or effective degrees of freedom cannot eliminate or penalise spurious parameters which are ill determined by the data. It might be dubious to invoke Occam's principle once more (after it has already been incorporated in the model evidence) to decide between models and only additional data should be used for final clarification. For purely pragmatic reasons of convenience one might still accept the model with formally fewer parameters even though it has the same model evidence and same effective degrees of freedom as a more complex one.

### Frequentist inference

Parameter estimation by maximising the likelihood of *θ *in equation 1 is equivalent to least squares (LS) optimisation, minimising the sum of squared errors(7)

An unbiased estimator  for the noise variance is(8)

Confidence intervals for the *p *estimated parameters are obtained from the covariance matrix of . This matrix is approximated by the Hessian matrix *H*(), that is, the matrix of second derivatives of the optimised function. The matrix is evaluated at . 95% confidence intervals for the *j*-th parameter (with *j *= 1, ..., *p*) are obtained as  ± 1.96 SE_*j*_, where each particular standard error (SE_*j*_) is obtained from the *j*-th diagonal element of the SE matrix(9)

### Model selection as hypothesis testing

A commonly used method for frequentist model comparison is a likelihood ratio test (LRT), in which two nested models with different number of parameters are compared. According to the null hypothesis, a simple model *M*_*s *_(with *p*_*s *_parameters) is correct, and thus the additional parameters in the more complex model *M*_*c *_with *p*_*c *_parameters are unnecessary. A *p*-value is obtained as tail probability of a *χ*^2 ^distribution with *p*_*c *_- *p*_*s*_ degrees of freedom of the statistic(10)

Applicability of an LRT is limited due to the requirement that the models are nested, and that the parameters are fully identifiable. Akaike's Information Criterion (AIC) allows ranking models even when they are nonnested. It is defined by(11)

where *p*_*i *_is the number of parameters in model *i *and *θ*_ML _is the value of *θ *that maximises the likelihood in 1. Standard tables exist for assessing the significance of AIC values [22].

### Implementation

MCMC methods are generic approaches to obtain samples from posterior distributions without the need to calculate the model evidence in equation 2 (for details see [16]). In the following we use MCSim, an MCMC simulator for differential equations developed mainly for application to pharmacokinetic models [23] for the estimation of posterior distributions and model probabilities. Essentially, at each step of the MCMC simulation, a set θ* of new parameters is chosen from a distribution centered on the current parameters *θ *(a multivariate Gaussian in MCSim). The differential equation is solved using the new parameters θ*. In the case of a symmetric proposal distribution, θ* is accepted and *θ *set to θ* with probability(12)

Five parallel MCMC chains were run for each model. Each chain consisted of 40,000 iterations (20,000 iterations in the simple one-equation model shown in [Additional file 1]). The first 20,000 samples (10,000 samples in the simple one-equation model) of each chain were discarded and then one every 10 iterations was stored. Convergence was assessed by applying the  statistic described in [16] to the five parallel runs. This statistic was below 1.05 in all estimations, except otherwise stated, indicating good convergence behaviour. Evaluation of one model takes a few minutes on a standard desktop machine. Monitoring and analysis of MCSim runs was performed with the statistical R software [24]. Maximum likelihood values were obtained by taking the highest value from the MCMC runs.

## Results

Here we present the model comparison results for different motif architectures (section Common network motifs), and for different parameterizations of the same FF motif (section Variants of feed forward motif). In each case, the models are introduced first, and the statistical comparisons described secondly in terms of Bayesian model evidence, DIC, effective degrees of freedom, and the maximum likelihood value from the MCMC chains. Statistical comparisons are here supported with the use of simulated data. This step is fundamental since it allows us to evaluate the performance of each approach before applying it to experimental data.

### Common network motifs

#### Models

**Figure 1:**
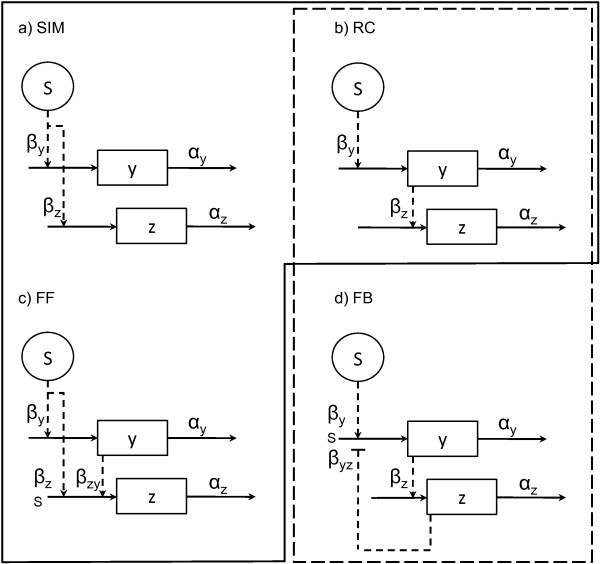
**Network motifs**. a) Single input motif (SIM), b) regulatory chain motif (RC), c) feed forward motif (FF), and d) feedback motif (FB). The abbreviations are: *S*, input signal; *y *and *z*, monitored variables; *α*, *β *reaction rates. Solid arrows denote production and degradation reactions, while dashed arrows denote control mechanisms. Normal arrowheads denote activation, while flat arrowheads denote inhibition.

We analyze the identifiability of the four motif architectures shown in figure 1, which have been found in transcription networks [1,2], based on simulated time series data from each of them. These motifs consist of one experimentally controlled input variable *S *and two state variables *y *and *z*. Briefly, the single input motif (SIM) involves a signal which simultaneously affects two targets, possibly with different strengths. The effect of a transcription factor upon the sequential expression of its target genes serves to illustrate this motif [25]. Regulatory chains (RCs) comprise a series of chained reactions, in which the end product of a reaction activates the next, as in the yeast cell cycle [1]. In feed forward loop motifs (FF), a master regulator controls an intermediate regulator, and both control a target component. Such an architecture has been shown to display many interesting roles in bacteria [26]. Finally, the feedback (FB) motif involves stimulation of a reaction by a signal, followed by end-product regulation (positive or negative) of the process. Negative feedback is known to control, for example, the DNA-damage response through p53 and Mdm2 in mammals [27].

These motifs can be expressed mathematically in many forms. As a first approximation, they are described here as systems of first order ordinary differential equations (ODEs), like those shown in table 1. Note that models SIM and RC are nested within model FF (for example, setting *β*_*zy *_to zero in model FF renders it the same as model SIM), thus they have been boxed together. The same phenomenon is observed between models RC and FB. The same model architectures described assuming cooperative production functions are given in [Additional file 1: supplementary table S4].

**Table 1:** Motif models.

motif	model
SIM	= *β*_*y*_*S *- *α*_*y*_*y*
	= *β*_*z*_*S *- *α*_*z*_*z*

RC	= *β*_*y*_*S *- *α*_*y*_*y*
	= *β*_*z*_*y *- *α*_*z*_*z*

FF	= *β*_*y*_*S *- *α*_*y*_*y*
	= (*β*_*zs*_*S *+ *β*_*zy*_*y*) - *α*_*z*_*z*

FB	
	= *β*_*z*_*y *- *α*_*z*_*z*

Simple ODE models for the network motifs of figure 1.

#### Analysis

Time series data were simulated from each of the models in tables 1 and [Additional file 1: supplementary table S4]. Parameter values were S = {0, 1, 2, 0} at times t = {0, 2, 6, 10}, and *β*_*y *_= *α*_*y *_= 1. Following [28], the coefficient and activation threshold in Hill functions were set to *h *= 2 and *θ *= 0.5, respectively. These are not experimentally determined parameter values. They are values required to yield biologically plausible solutions, a strategy also applied in [4]. Finally, 30 equally spaced data points were sampled from each time course, and a Gaussian error term with mean 0 and standard deviation 0.05 was added to simulate measurement errors.

The choice of parameter priors is critical in Bayesian model comparison. To make comparison as fair as possible, the same distribution was chosen for all rate constants in the simulations below, namely a log-normal distribution with mean 0 and standard deviation 1 in log-space. Finally, for the prior on the noise variance *σ*^2 ^an inverse Gamma distribution with shape *a *= 0.5 and scale *b *= 0.05 was chosen (a fairly broad and heavy-tailed prior, the mean does not exist and the mode is at 0.1/3).

Table 2 provides a ranking of the four candidate models (SIM, RC, FF, FB in columns), given data generated from each of the four models (in rows). The analysis of the SIM and FF models, given that the data are generated by the SIM model, is of high interest since these models are nested. As shown in the first row in table 2, both models result in a similar model evidence (around 89), that is, the fit by FF is as good as the one by SIM. Note that FF has one more parameter than SIM. By looking at the effective degrees of freedom, we see that the additional parameter in FF is not estimated. Since model SIM has fewer formal parameters, a pragmatic approach would prefer model SIM. In this test, the maximum likelihood values render the SIM, RC and FF models almost equally plausible. A likelihood ratio test between SIM and FF yields  = 0.92 which is not significant at the 5% level and we reject the alternative hypothesis that the additional parameter in model FF is necessary. The LRT cannot be applied to assess the SIM/RC pair because the models are not nested. In this case, the conclusion derived by looking at AIC is that neither model can be discarded.

**Table 2:** Model comparison results from simple network motifs.

data source	measure	SIM	RC	FF	FB
SIM	log *p*(*Y *| *M*_*i*_)	**89.3**	73.74	**89.6**	49.03
	DIC	**-198.1**	-192.5	**-198.8**	-177.51
	pD	3.93	2.94	4.01	4.34
	log *p*(*Y *| , *M*_*i*_)	**102.99**	**102.44**	**103.45**	100.70
	AIC	**-197.98**	**-196.88**	**-196.9**	-191.4

RC	log *p*(*Y *| *M*_*i*_)	29.21	**87.61**	73.58	55.38
	DIC	-86.17	**-194.60**	-187.13	-175.21
	pD	4.08	3.92	4.53	4.66
	log *p*(*Y *| , *M*_*i*_)	47.18	**101.22**	100.46	97.62
	AIC	-86.36	**-194.44**	-190.92	-185.24

FF	log *p*(*Y *| *M*_*i*_)	80.20	57.60	**93.43**	22.95
	DIC	-184.7	-153.1	**-208.8**	-131.53
	pD	4.06	3.92	4.81	5.01
	log *p*(*Y *| , *M*_*i*_)	96.42	81.03	**109.17**	77.64
	AIC	-184.84	-154.06	**-208.34**	-145.28

FB	log *p*(*Y *| *M*_*i*_)	-17.60	-13.93	-39.68	**79.07**
	DIC	2351.3	2718.1	2375.8	**-181.37**
	pD	4.04	3.66	4.61	4.98
	log *p*(*Y *| , *M*_*i*_)	-1171.59	-1355.33	-1176.64	**95.62**
	AIC	2351.2	2718.66	2363.26	**-181.24**

Model comparison results for artificial data from the simple ODE models SIM, RC, FF (type 1 coherent with OR gate) and negative FB motifs. Each fit is assessed in terms of model evidence, log p(Y | M*i*), the deviance information criteria, or DIC, the effective degrees of freedom, or pD, the maximum likelihood value obtained from MCMC simulations, log p(Y | , *M_i_*), and Akaike's Information Criteria, or AIC.

Bayesian model evidence values clearly favour the true model when data is generated from the RC motif (second row in table 2). Note that the maximum likelihood also favours the correct option, although here the relative difference between RC and FF is smaller than the differences in model evidence. When data from the more complex models FF and FB are used (third and fourth rows), the models that achieve the best fit are the true models FF and FB respectively, no matter the statistical measure chosen. Note that the effective degrees of freedom are close to the correct number of five for the FF data as opposed to four for the SIM data when fitting the FF model.

We performed the same analysis assuming the motif models could be defined with cooperative production terms (see [Additional file 1: supplementary table S4]). Bayesian model evidence always favours the correct model ([Additional file 1: supplementary table S5]). The results from of the AIC are less conclusive, which favours the wrong model (FB) in at least one case (RC).

### Variants of feed forward motif

#### Models

**Figure 2:**
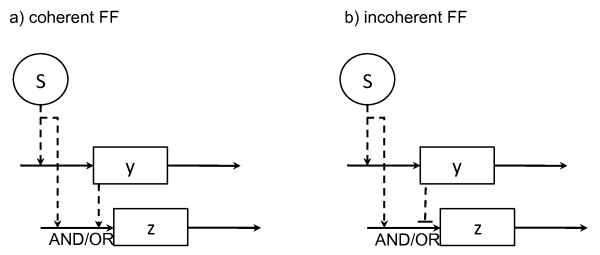
**Feed forward motif subtypes: coherent and incoherent**. In a feed forward (FF) motif, the interaction between the *master *and *intermediate *regulators (named *S *and *y*, respectively) modulates the response of the *target *component, *z*. In a type 1 coherent FF motif (a), both *S *and *y *are activating signals, while *y *is a repressing signal in a type 1 incoherent FF motif (b). Other --less frequent-- subtypes have been reported [28].

The generic FF architecture shown in figure 1c has been found to exist in different forms depending on the signs in the intermediate and main branches. The abundance of these different FF subtypes has been studied in *E. coli *and yeast [28,29]. It has been shown that coherent type 1 FF motifs (FF.C1), where the signal is activating in both branches, and incoherent type 1 FF motifs (FF.I1), which features negative regulation from *y *to *z*, are the most frequent (figure 2).

In [28] it is shown through theoretical mathematical modeling that these FF forms display specific dynamic behaviours. In their models, the rates of change of the concentration of the state variables *y *and *z *are expressed as a combination of a nonlinear production term and a linear degradation term (basal production rates are set to zero). Following [28] and the notation in figure 2, we have that the rate of change of *y*, as determined by an activating signal *S *with activation threshold *θ*_*Sy *_and Hill coefficient *h*, is defined as(13)

where *f*^+^(*S*, *θ*_*Sy*_, *h*) = *S*^*h*^/( + *S*^*h*^). If S were a repressor, the associated Hill function would be *f*^-^(*S*, *θ*_*Sy*_, *h*) = /(+*S*^*h*^). The rate of change of *z *is generically defined as(14)

where *g *describes the function (or gate type) that integrates the signals from *S *and *y *on the promoter of *z *[28]. For an AND gate integrating two activating signals, we have *g *= *f*^+^(*S*, *θ*_*Sz*_, *h*)*f*^+^(*y*, *θ*_*yz*_, *h*), while for an activating and a repressing signal, we have *g *= *f*^+^(*S*, *θ*_*Sz*_, *h*)*f*^-^(*y*, *θ*_*yz*_, *h*).

FF behaviours have been experimentally explored for the coherent (AND gate), coherent (OR gate) and incoherent (AND gate) subtypes in the arabinose, flagella, and galactose systems of *E. coli *respectively [5-7]. Interestingly, the time series data available allows following the FF component *z *(figure 2) as the system is switched ON and OFF, in comparison to a non-FF control system. Below we present a description of each control and FF instance, and a formal statistical analysis of the data.

#### Arabinose system

**Figure 3:**
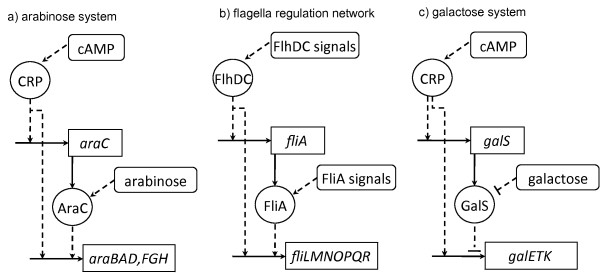
**Bacterial feed forward systems**. The bacterial feed forward systems analysed here are the arabinose system (a), the flagella network (b), and the galactose system (c). Figures adapted from [5-7].

The arabinose (ara) system is the set of genes that allows intake of the sugar arabinose (figure 3a). These genes, which encode catabolism (AraBAD) and transport (AraFGH) proteins, are activated in the presence of arabinose provided their preferred carbon source--glucose--is not available. That is, two signals regulate the system: absence of glucose *and *presence of arabinose, and two different transcription factors respond to them: i) CRP, that becomes an activator upon sensing the no-glucose signal cAMP, and ii) AraC, that acts as a transcriptional activator upon binding arabinose. CRP promotes the expression of *araC*, and both CRP and AraC promote the expression of the *araBAD/FGH *genes [5].

Here we test whether the generic FF.C1.AND model described in [28] can be used to model the ara system. This is defined by:(15)

where AraC is abbreviated as AC, and the product of gene *araBAD *is abbreviated as AB. Since experiments were performed under saturating arabinose levels, all the AraC protein produced is assumed to be active. Experiments also revealed that the basal rates of AC and AB production, *B*_*ac *_and *B*_*ab*_, are small compared to the levels reached upon activation [5], thus they are set to zero in the parameter inference exercise. The ON step consists of a constant signal, CRP = 1, and initial conditions AC(0) = 0 and AB(0) = 0, while the OFF step is defined as CRP = 0, and AC(0) = 1, AB(0) = 1.

#### Control model for arabinose system

In [5] the dynamic behaviour of the coherent FF motif was compared with a simpler non-FF regulation motif in which two unlinked TFs regulate a common target via an AND gate, the *control *system ([Additional file 1: supplemental figure S3a]). The module shares the main TF with the arabinose system, CRP. The other positive regulation flow consists of a double-repression mechanism: the *lacZ *repressor, LacI, is repressed by lactose or IPTG. In other words, lactose (or IPTG) allows transcription from *lacZ*. Since the two inputs to the system, CRP and lactose, are independent and externally controlled, a model with one single equation is suggested here. This is defined as:(17)

This control model is set up as follows: an ON step consists of CRP = 1, lactose = 1, LZ(0) = 0, and an OFF step consists of CRP = 0, lactose = 1, LZ(0) = 1. As before, *B*_*lz *_was assumed to be 0. In terms of the target component *z*, the only difference between this control model and a coherent FF motif is the fact that the two *z *inputs are independent.

#### Flagella system

The flagella biosynthesis network of *E. coli *is the system that governs the swimming capabilities of the bacteria, in such a manner that it moves away from its current location when growth conditions become mildly unfavourable. As illustrated in figure 3b, the genes that produce the flagella motor are regulated by a FF motif (see [6] and references therein). The components of this motif are the master regulator FlhDC (*S*), the intermediate regulator FliA (*y*), and a target operon (*z*) composed of a series of genes, *fliLMNOPQR *(hereafter abbreviated *fliL*). The two regulators are activators, thus forming a coherent FF, that converge upon *fliL *with OR input logic [30].

An initial candidate model for the flagella network, model FF.C1.OR.1, is based on the FF.C1.OR equations described in [28]:(18)

where FlhDC is abbreviated as FD, FliA as FA, and the product of gene *fliL *as FL. Under this model, an ON step is simulated by FD = 1, FA(0) = 0, and FL(0) = 0, while an OFF step is set up as FD = 0, FA(0) = 1, and FL(0) = 1.

#### Control model for flagella system

In [6] the kinetic activity of this module was compared to that of a differently engineered version of the system in which the gene for *fliA *was deleted. This *control *system, which is a *simple regulation *motif [26], only contains two elements: the positive regulator FlhDC, and its target operon *fliL *([Additional file 1: supplemental figure S3b]). The corresponding model consists of a linear function-regulated *z *in which no *cis*-regulatory function is needed because the promoter is controlled by one input only:(20)

The initialization conditions are FD = 1, FL(0) = 0 for the ON step, and FD = 0 and FL(0) = 1 for the OFF step.

#### Additional flagella models

We consider two alternative models for the flagella FF motif incorporating time delays. The interval needed for FliA activation is explicitly modeled via a time delay, *τ*, in model FF.C1.OR.2(21)

In this type of models, delay differential equation models, the concentration of FA used in the equation at time *t *is the one which existed earlier at time *t *- *τ*, where *τ *is an additional parameter that needs to be estimated. Intuitively, while the sign-sensitive delay behaviour is well explained by the type of logic gate used, only the incorporation of a time delay on *y *could explain the observed *increase *in the concentration of *z *upon an OFF signal step (see time series in [6]). This model uses the same initialisation conditions described for model FF.C1.OR.1.

Finally, a last model for the flagella system was tested in which the time delay affects both the expressions for FA and FL, model FF.C1.OR.3:(22)

#### Galactose system

In an incoherent FF motif, the two regulation paths flowing from the master regulator display opposite signs. In particular, in a type 1 incoherent FF motif, the branches from *S *to *y *and *S *to *z *are activating, but the intermediate branch from *y *to *z *is repressing (figure 2b). In [7] the dynamics for a FF.I1.AND motif were studied in *E. coli *cells *in vivo *in the network of genes in charge of galactose (gal) metabolism (figure 3c). Similarly to the ara system, the gal system is activated in the absence of glucose. Under these conditions, CRP simultaneously activates the *galETK *operon and the *galS *gene. GalS is a repressor of *galETK *that unbinds from the target promoter upon galactose binding. Thus, in contrast to the ara system, an ON step here consists in sensing the no-glucose signal cAMP in absence of the alternative sugar galactose. In the presence of galactose, the system becomes a coherent FF motif, reaching the maximal rate of expression of the *galETK *operon. According to [7], the biological explanation behind this counter-intuitive effect may be that cells prepare to use galactose as soon as they run out of glucose. The galactose catabolism machinery is produced at medium levels, allowing fast use of this alternative carbon source as soon as it becomes available, in which case the gal system is maximally activated.

The equations suggested here to model the gal system are(24)

where GalS is abbreviated as GS, and the product of gene *galETK *as GE. An ON step is set up by CRP = 1, GS(0) = 0, GE(0) = 0, while no OFF step was simulated because it was not provided in the original paper [7]

#### Control model for galactose system

The same control module developed for the arabinose system was used as a control for the gal system in [7], with the difference that no repressor inhibitor was utilised. Thus, it resulted in a multiple input module that integrated two incoherent signals: activation from CRP and inhibition by LacI ([Additional file 1: supplemental figure S3a]). The control model therefore consists of(26)

where LI refers to the repressor LacI. The simulation conditions are CRP = 1, LI = 1, LZ(0) = 0.

#### Analysis

Here we first explore the different FF motif subtypes using simulated data generated from equations 15, 18 and 24 under the same conditions used in the experiments: the same signal function, number of observed variables and dataset size. The latter implies that the number of data points for each of the FF systems is: 60 for the arabinose system (30 in ON, 30 in OFF step), 37 for the flagella system (20 in ON, 17 in OFF step), and 15 for the galactose system (only ON step). Note that only one variable -*z*- is monitored here, and *y *is set as a hidden variable. The parameters were set to the same values as indicated in the theoretical work by [28], and parameter priors were the same as indicated in section Analysis.

As shown in table 3, the simpler control models perform poorly, as expected. They might therefore be used as control systems to demonstrate that the specific dynamic signature found in time series data from an FF system cannot be reproduced by simpler network architectures. Here we take the analysis one step further and compare the identifiability of the different FF subtypes as well. When the data are generated from the FF.C1.AND (first row in table 3) or the FF.C1.OR.1 (second row) motifs, the correct model is identified using any of the model comparison methods: model evidence, DIC or maximum likelihood. However, for the FF.I1.AND case (third row), the correct model is only slightly favoured over the competing FF.C1.OR.1 model according to all methods tested.

**Table 3:** MCMC model comparison results for the artificial FF datasets.

data source	measure	CONTROL	FF.C1.AND (Eqns 15)	FF.C1.OR.1 (Eqns 18)	FF.I1.AND (Eqns 24)
		(Eqn. 17)			
FF.C1.AND (Eqns 15)	log *p*(*Y *| *M*_*i*_)	85.98	**108.94**	106.51	86.53
	DIC	-217.98	**-388.91**	-292.81	-205.64
	log *p*(*Y *| *θ*_*ML*_, *M*_*i*_)	111.22	**165.18**	164.34	111.15
	AIC	-208.44	**-316.36**	-314.68	-208.3

FF.C1.OR.1 (Eqns 18)	log *p*(*Y *| *M*_*i*_)	(Eqn. 20) 40.12	30.90	**48.09**	42.55
	DIC	-108.96	-102.47	**-136.65**	-117.22
	log *p*(*Y *| *θ*_*ML*_, *M*_*i*_)	62.73	60.06	**86.94**	69.97
	AIC	-111.46	-106.12	**-159.88**	-125.94

FF.I1.AND (Eqns 24)	log *p*(*Y *| *M*_*i*_)	(Eqn. 26) 12.52	8.28	**13.78**	**14.02**
	DIC	-38.33	-35.75	-36.75	**-41.63**
	log *p*(*Y *| *θ*_*ML*_, *M*_*i*_)	26.66	25.69	**30.09**	**30.86**
	AIC	-39.32	-37.38	**-46.18**	**-47.72**

Datasets have the same number of samples as the experimental data from [5-7]. They were generated using Equations 15 (first row), 18 (second row) and 24 (third row). Note that the model labelled *control *is specific for each dataset: ara control (Equation 17) on the first row, flagella control (Equation 20) on the second row, and gal control (Equation 26) on the last row.

**Table 4:** MCMC model comparison results for the experimental FF datasets.

data source	measure	CONTROL	FF.C1.AND	FF.C1.OR.1	FF.I1.AND
ara system	log *p*(*Y *| *M*_*i*_)	51.22	**74.99**	72.31	51.88
	DIC	-158.69	-435.02	**-445.81**	-275.31
	log *p*(*Y *| *θ*_*ML*_, *M*_*i*_)	83.99	140.05	**147.06**	83.99
	AIC	-157.98	-264.10	**-278.12**	-151.98

flagella system	log *p*(*Y *| *M*_*i*_)	**-16.22**	-264.64	-73.81	*-∞*
	DIC	-54.99	**-41275.70**	-2296.40	5878.25
	log *p*(*Y *| *θ*_*ML*_, *M*_*i*_)	**31.02**	-15.28	-4.72	-256.50
	AIC	**-52.04**	46.56	25.44	529.20

gal system	log *p*(*Y *| *M*_*i*_)	-8.71	-13.29	-4.49	**-0.42**
	DIC	10.53	9.88	-3.53	**-20.47**
	log *p*(*Y *| *θ*_*ML*_, M_*i*_)	-1.79	-1.13	11.57	**12.53**
	AIC	13.58	18.26	-7.14	**-9.04**

The equations used are the same as in table 3.

**Figure 4:**
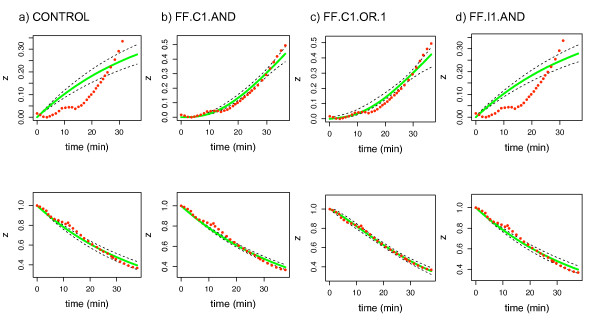
**Reconstructing the arabinose dataset**. Model predictions for the arabinose dataset, using the posterior parameter values inferred with MCMC. The fractional value of the feedforward element *z *(target gene AB in the arabinose model) is shown as a function of time. Red dots denote the original data as provided in [5], dashed black lines the 95% credible interval, and the average solution is shown in green. The first row shows the ON steps, while OFF steps are given in the second row.

Table 4 shows the results of comparing real experimental data from the *E. coli* systems with the feed forward models. In the case of the ara system, model evidence identities FF.C1.AND as the underlying model, which agrees with current knowledge about the system [5]. However, DIC and maximum likelihood favour the FF.C1.OR.1 model. Figure 4 shows the corresponding model reconstructions, that is, the predicted solution under each tested model. Although the predictions from models FF.C1.AND and FF.C1.OR.1 (Figures 4a and 4c) appear very similar at first glance, note that the 95% confidence interval for the latter is slightly wider. Therefore, in agreement with table 4, the best match between data and prediction is obtained for the correct model (Figure 4b).

In the case of data from the flagella network, the results unexpectedly favour the control model. Since this network is indeed believed to be composed of a feed forward FF.C1.OR motif [6], additional modifications of this motif were tested (models FF.C1.OR.2 and 3) in order to improve the fit. The results are shown in table 5. Again the statistical methods disagree, with the Bayesian model evidence still favouring the simple control model, while the AIC prefers FF.C1.OR.3. This discrepancy might be explained by the tendency of Bayesian methods to favour simpler models. Overall, it seems that if an FF.C1.OR type model fits the flagella data at all, it has to be a more complex one than for the other two systems, possibly involving delays. This hints at the possiblity of the essential involvement of further elements not represented in the current system.

**Table 5:** Assessment of additional flagella models for the flagella dataset [6], with MCMC.

Measure	CONTROL	FF.C1.OR.1	FF.C1.OR.2	FF.C1.OR.3
log *p*(*Y *| *M*_*i*_)	**-16.22**	-73.81	-179.11	-33.81
DIC	-54.99	**-2296.40**	23.59	-212.89
log *p*(*Y *| *θ*_*ML*_, *M*_*i*_)	31.02	-4.72	30.38	**35.09**
AIC	-52.04	23.44	-46.76	**-54.18**

The prior for the delay parameter was a Normal distribution with mean and variance 10, truncated at [0,100].

Finally, in the case of data from the *gal *system, all approaches agree that the data are drawn from a FF.I1.AND model (the Bayes factor of FF.I1.AND to model FF.C1.OR.1 is 58.55, which can be interpreted as decisive evidence in favour of the incoherent FF motif according to [21]), which agrees with current knowledge [7].

## Discussion

When building a model for a biological system, one has to decide whether to use parameter values from the literature or estimate them from the data. While the first option may be very useful, one has to bear in mind the limitations that extrapolating parameter values from other systems and experimental conditions have [31]. Thus, when data is available for the system under study, parameter inference becomes an interesting strategy. However, knowledge about biological systems is often sparse, and thus more than one model structure is often compatible with the system of interest. That is, more than one model structure could potentially be calibrated. In order to explore which model is preferable given the available data, a natural step after parameter inference consists in formally assessing the validity of all possible -now parameterized- candidate models. Despite its importance, this step is often overlooked.

Here we have provided a short overview of the Bayesian and frequentist approaches to model comparison. Then, we have applied the model comparison techniques to two cases. First, we have investigated the identifiability of a series of transcription regulation motif architectures (SIM, RC, FF and FB). The objective was to find out if one could infer the correct underlying model structure given time series data from each of these motifs (tables 2 and [Additional file 1: supplementary table S5]). For the case of the nested models, SIM and FF, when the data were generated by the SIM model, models SIM and FF have about equal model evidence. This is expected since the FF model can mimick the SIM model and the additional parameter can not be estimated from the data. From a pragmatic point of view one might accept the SIM model in this case. Note that AIC failed to identify the correct model in this case. For all the other datasets, model evidence, DIC and AIC favoured the correct model.

Secondly, it is known that the same model architecture can give rise to different dynamics depending on the particular model parameterization. To explore this issue, we have focused on the FF motif, an architecture for which extensive experimental caracterization has been carried out during the past years [5-7], following the description of different FF subtypes (figure 2) with different dynamic properties [28]. In [5-7] it is shown that the predicted behaviour of FFs is indeed observed *in vivo*. That is, their functionality is conserved even when they are embedded in large genetic systems. To address the question whether the biological signals are strong enough for the specific type of model parameterization to be identified from experimental data, we have analyzed such data under a series of candidate FF subtypes.

The motivation behind the original papers [5-7] was to compare each FF subtype to its non-FF control in a qualitative manner. We have formalized this comparison and have taken the analysis one step further discriminating each FF subtype from the others. Analysis of artificial data indicates that the experiments should be informative enough for the coherent FF subtypes to be differentiated from their controls, but also from each other. Identification of the incoherent subtype seems to be harder than the rest given the available data (table 3). Comparison of each FF model with its corresponding control model given the experimental datasets (table 4) shows that the Bayesian framework agrees with the conclusions that Alon and colleagues derived from visual inspection of the plots in all but the flagella network case [5-7].

While the *cis*-regulatory functions involved in the flagella gene network are known [30], no mathematical model is given in [6] to describe the experimental data corresponding to this system. Flagella system models including time delay effects have been defined here based on current biological knowledge. Bayesian model evidence still points towards the simpler control model without an additional FF branch as the best explanation for the data, but the AIC indicates that a delay model might be plausible. The importance of a delay element hints towards involvement of further unobserved components in the motif.

It could be argued that the body of information assumed available to generate the dataset used is so large that no model uncertainties remain. We wish to stress that embarking on a model comparison exercise is a way to make sure that all relevant mechanisms have been accounted for. Therefore, model comparison strategies should be regarded as *complementary to *and *dependent on *experimental work, rather than as standalone techniques.

## Conclusions

We have given an overview of model comparison methods suitable for selecting a plausible network motif structure among a set of candidate models for time series data on gene regulation. We show that it is practical to apply maximum likelihood as well as Bayesian model comparison procedures to test ideas about underlying mechanisms of biological pathways in a formal and quantitative way.

## Authors' contributions

All authors participated in the design of the study, helped to draft the manuscript. NDP performed the statistical analysis and mainly drafted the manuscript. LW coordinated the project. All authors read and approved the final manuscript.

## Supplementary Material

Additional file 1 **Supplementary material for "Statistical model comparison applied to common network motifs"**. The example of estimating parameters for a simple DE model consisting of one equation is used to exemplify and discuss statistical issues of model selection as clearly as possible. Results for extended cooperativity effects in models SIM, RC, FF, and FB are also shown. Click here for file
