# Supplementary material for: Statistical model comparison applied to common network motifs
Source: BMC Syst Biol. 2010 Mar 3;4:18. doi: 10.1186/1752-0509-4-18 (PMC2855527; doi:10.1186/1752-0509-4-18)
Supplement: Additional file 1 — Supplementary material for "Statistical model comparison applied to common network motifs". The example of estimating parameters for a simple DE model consisting of one equation is used to exemplify and discuss statistical issues of model selection as clearly as possible. Results for extended cooperativity effects in models SIM, RC, FF, and FB are also shown. [file 1752-0509-4-18-S1.pdf]

Supplementary material for "Statistical model comparison applied to common network motifs"

Núria Domedel-Puig*[1] , Iosifina Pournara[2] , Lorenz Wernisch[3]

[1]Departament de Física i Enginyeria Nuclear, Universitat Politècnica de Catalunya, Edifici GAIA, Rambla de Sant Nebridi s/n 08222, Terrassa, Barcelona, Spain
[2]School of Crystallography, Birkbeck College, University of London, Malet Street, London WC1E 7HX, UK
[3]MRC Biostatistics Unit, Robinson Way, Cambridge CB2 0SR, UK

Email: Núria Domedel-Puig*- nuria.domedel@upc.edu; Iosifina Pournara - i.pournara@cryst.bbk.ac.uk; Lorenz Wernisch - lorenz.wernisch@mrc-bsu.cam.ac.uk;

*Corresponding author

Simple one-equation model

In order to explore some issues of parameter inference and model comparison, we start with a simple model $\mathcal{M}_1$ of a single dependent variable y, representing the concentration of some chemical species

$$dy/dt = \beta_y S - \alpha_y y \tag{1}$$

where β_y is the production rate, S a signal, and α_y the degradation rate.

The choice of time points where data are sampled is critical for dynamic systems. If data are sampled from the steady state $y_s = \beta_y S/\alpha_y$ only, an identifiability problem arises. Since the time scale is lost, we can multiply and divide the parameters β_y and α_y by a single factor and obtain the same solution. In order to avoid this situation, it is important that data are sampled before the system reaches a steady state. Indeed, figures S1a to S1c illustrate that data obtained from a complete series, y_c, are more informative than data obtained by the steady state interval alone, y_{ss}, assuming a constant signal $S = 1$. In fact, the actual values of β_y and α_y are irrelevant when using y_{ss} data as long as the ratio β_y/α_y remains the same.

We have also explored the case when the input signal is set to the values 1, 2 and 0 at times 0, 4 and 7 in a stepwise manner. The more diverse the input function, the narrower the posterior distribution becomes (figure S1d). This is caused by the known added variability in the input, which partially determines the values of the parameters. Similarly, increasing the number of sampled time points has a profound effect on the posterior distribution. When comparing a dataset of 11 time points with one of 40, the posterior

variance of β_y decreases from 0.01 to $4.67 \cdot 10^{-4}$ (see table S1).

Noise levels

Assuming that the measurements of the system have Gaussian noise with variance σ^2, each observation y_{obs} is normally distributed

$$y_{\mathrm{obs}} \sim \mathcal{N}(y(\theta), \sigma^2) \tag{2}$$

where $y(\theta)$ is the observed variable—dependent on parameters $\theta = \{\beta_y, \alpha_y\}$—free of noise. We explore two cases: one where the noise variance is assumed to be known, and one where it is unknown and has to be estimated as an additional model parameter. Using data generated from equation 1 with true noise variance equal to $\sigma^2 = 0.04$, we observe that if the variance is fixed at a smaller value than the true one (here we use $0.01 < \sigma^2$), the posterior distribution of the rate parameters is overly optimistic. If the variance is assigned a value higher than the true one (we use $1 > \sigma^2$), the posterior becomes very vague. The best results are obtained when the variance is set to its true value during the inference procedure, followed by the results obtained when it is estimated as an additional parameter (figure S2). In other words, here we explore the effect of either fixing or estimating the noise variance. If available, such variance should be fixed to its known value or assigned a highly informative prior. This simplifies the inference exercise by providing more initial information (reducing the number of unknown parameters or restricting the space of possible values). However, in the case where the exact value of the noise variance is unknown, we conclude that it is advisable to estimate it rather than to fix it to some arbitrary value.

Prior Specification

We investigate the sensitivity of the rate parameters inference to prior distributions using different uniform distribution intervals on the priors for β_y and α_y (for the noise variance, we set a constant inverse Gamma prior with shape and scale parameters of 0.5 and 0.05, respectively). Table S2 shows that even when a very uninformative prior is used, that is, a distribution spanning a large interval like that in the fourth row, the posterior distributions of the parameters are still centered around the true values. This shows that in this case –where samples are taken from the entire time series– the data is informative enough to drive the inference process, irrespective of the uncertainty in the prior density. Table S2 also lists the parameter estimates derived with standard least squares optimization (in this case, the Nelder and Mead method). In particular, the algorithm was started on 100 random initial parameter values drawn from a Normal distribution with mean and variance 1.

Model Comparison

We investigate the ability of the Bayesian and frequentist frameworks to identify the correct option between two competitive candidate models under different noise levels. We use $\mathcal{M}_1$, the model of equation 1, and a second model $\mathcal{M}_2$ that contains a degradation term with a bimolecular reaction:

$$dy/dt = \beta_y S - \alpha_y y^2 \tag{3}$$

The data are generated using model $\mathcal{M}_1$, and the added noise is sampled from a white Gaussian distribution with variance $\sigma^2 = \text{var}(y)/\text{SNR}$. Note that var($y$) is the variance of the data free of noise, and the signal-to-noise ratio (SNR) is set at different values between 0.5 and 100.

Table S3 shows the results obtained. The interpretation of Kass and Raftery (1995) of the Bayes factor indicates that for SNR > 1 there is a decisive evidence in favour of the true model $\mathcal{M}_1$, for SNR equal to 1 there is a substantial evidence in favour of $\mathcal{M}_1$, and there is no evidence for either model for extensively large noise levels (SNR equal to 0.5).

Table S3 also shows the log likelihood computed by the optimisation method of Nelder and Mead, $p(y|\theta^{ls}_{ML}, \mathcal{M})$. The AIC derived from these values favour the true model ($\mathcal{M}_1$) in all cases but the last two, where there is no evidence to discard neither model. The figures in parentheses show the highest value of the likelihood function sampled during the MCMC runs, that is, the maximum likelihood value, $p(y|\theta^{mcmc}_{ML}, \mathcal{M})$. These values appear to be a reasonable approximation to $p(y|\theta^{ls}_{ML}, \mathcal{M})$, except for the first case. With respect to model performance, the trends observed here among the frequentist and the Bayesian ways of assessing model performance are the same: as the noise increases (lower SNR), the difference between models blurs.

n	$\hat{\beta}_y \vert y_c$	$\hat{\alpha_y} \vert y_c$
11	1.02 (0.01)	1.02 (0.01)
20	1.003 (0.002)	1.004 (0.0026)
30	1.004 (0.00092)	1.004 (0.001)
40	1.003 (0.00046)	1.003 (0.00053)

Table 1: Effect of dataset size on posterior distribution of rate parameters, given a Uniform prior U[0,3]. The values shown are the posterior median and its associated variance (in brackets). Note the true values are $\beta_y = \alpha_y = 1$.

prior	CI[$\hat{\beta_y}$]	CI[$\hat{\alpha_y}$]	CI[$\hat{\sigma}^2$]
U[0,2]	[0.807,1.367]	[0.741,1.293]	[0.011,0.068]
U[0,5]	[0.815,1.371]	[0.755,1.311]	[0.011,0.070]
U[0,10]	[0.818,1.387]	[0.754,1.322]	[0.011,0.074]
U[0,20]	[0.807,1.331]	[0.743,1.275]	[0.011,0.069]
ML	[0.860,1.128]	[0.793,1.045]	0.014

Table 2: *Parameter inference results for the simple one-equation model.* The first four rows show the posterior credible interval (CI) of the parameters obtained with the prior specifications indicated in the first column, as computed by MCMC. That is, the interval where the samples fall 95% of the times, given the posterior distribution for each parameter. The last row shows the results from maximum likelihood optimisation (equation 7 in main text), in particular, using the Nelder and Mead implementation available in the R statistical package.

SNR	$p(y\|\mathcal{M}_1)$	$p(y\|\mathcal{M}_2)$	BF	$p(y\|\theta^{ls}_{ML}, \mathcal{M}_1)$	$p(y\|\theta^{ls}_{ML}, \mathcal{M}_2)$	AIC($\mathcal{M}_1$)	AIC($\mathcal{M}_2$)
∞	4.32	-6.58	$5.42 \cdot 10^4$*	72.63(21.31)	2.68(2.42)	-139.26*	0.64
100	3.17	-6.04	$9.99 \cdot 10^3$*	16.75(16.46)	4.45(4.16)	-27.5*	-2.9
10	0.35	-7.81	$3.50 \cdot 10^3$*	9.74(9.73)	-0.38(-0.38)	-13.48*	6.77
1	-8.29	-9.90	5.00*	-3.43(-3.95)	-5.02(-4.97)	12.86	16.04
0.5	-18.57	-16.59	0.14	-13.56(-13.57)	-11.77(-11.79)	33.12	29.54

Table 3: *Effect of noise level on model identification.* The first column lists the signal-to-noise ratio. The next three columns show the Bayesian results, namely, the evidence for each model in logarithmic scale $\log p(y|\mathcal{M})$, and the associated Bayes factors (BF). The last four columns list the frequentist results. The log data likelihood computed using the least squares parameter estimates, $\log p(y|\theta^{ls}_{ML}, M)$, is given first. The values in brackets represent the highest value of the likelihood of each model sampled during the MCMC run in log scale, $\log p(y|\theta^{mcmc}_{ML}, M)$. Finally, the associated Akaike Information Criterion (AIC) values are given.

Cooperativity models

Cooperativity models are defined in table S4. Corresponding results are given in table S5.

motif	model
SIM	$\dot{y} = \beta_y f^+(\theta_{Sy}, S, h_{sy}) - \alpha_y y$
	$\dot{z} = \beta_z f^+(\theta_{Sz}, S, h_{sz}) - \alpha_z z$
RC	$\dot{y} = \beta_y f^+(\theta_{Sy}, S, h_{sy}) - \alpha_y y$
	$\dot{z} = \beta_z f^+(\theta_{yz}, y, h_{yz}) - \alpha_z z$
FF	$\dot{y} = \beta_y f^+(\theta_{Sy}, S, h_{sy}) - \alpha_y y$
	$\dot{z} = \beta_z(f^+(\theta_{Sz}, S, h_{sz}) + f^+(\theta_{yz}, y, h_{yz})) - \alpha_z z$
FB	$\dot{y} = \beta_y(f^+(\theta_{Sy}, S, h_{sy}) + f^-(\theta_{zy}, z, h_{zy})) - \alpha_y y$
	$\dot{z} = \beta_z f^+(\theta_{yz}, y, h_{yz}) - \alpha_z z$

Table 4: Cooperative ODE models for the network motifs.

data source	measure	SIM	RC	FF	FB
SIM	$\log p(y\|M_i)$	**71.87**	57.07	**72.84**	-203.36
	DIC	**-159.29**	-141.84	**-159.65**	-53.97
	pD	5.82	4.08	5.01	-221.36
	$\log p(y\|\theta_{ML}^{mcmc}, M_i)$	**85.45**	79.42	**85.41**	67.06
	AIC	**-162.9**	-146.4	-160.82	-120.12
RC	$\log p(y\|M_i)$	83.83	**84.02**	83.62	-152.19
	DIC	**-184.83**	-180.41	-184.29	274.03
	pD	5.19	4.64	4.71	4.91
	$\log p(y\|\theta_{ML}^{mcmc}, M_i)$	93.48	95.57	**98.36**	-128.21
	AIC	-178.96	-183.14	-186.72	**-270.42**
FF	$\log p(y\|M_i)$	94.79	71.61	**96.31**	-304.08
	DIC	-207.70	-175.55	**-208.86**	81.47
	pD	5.95	5.27	6.12	-42.07
	$\log p(y\|\theta_{ML}^{mcmc}, M_i)$	109.79	96.95	**110.64**	88.29
	AIC	**-211.58**	-181.9	-211.28	-162.58
FB	$\log p(y\|M_i)$	-705.70	-590.14	$-\infty$	**79.44**
	DIC	1343.98	1115.04	1188.39	**-186.59**
	pD	5.05	5.12	57.01	6.73
	$\log p(y\|\theta_{ML}^{mcmc}, M_i)$	-661.66	-548.35	-532.19	**100.11**
	AIC	1335.32	1108.7	1078.38	**-186.22**

Table 5: Model comparison results for artificial data from the cooperative version of SIM, RC, FF (coherent with OR gate) and negative FB motifs.

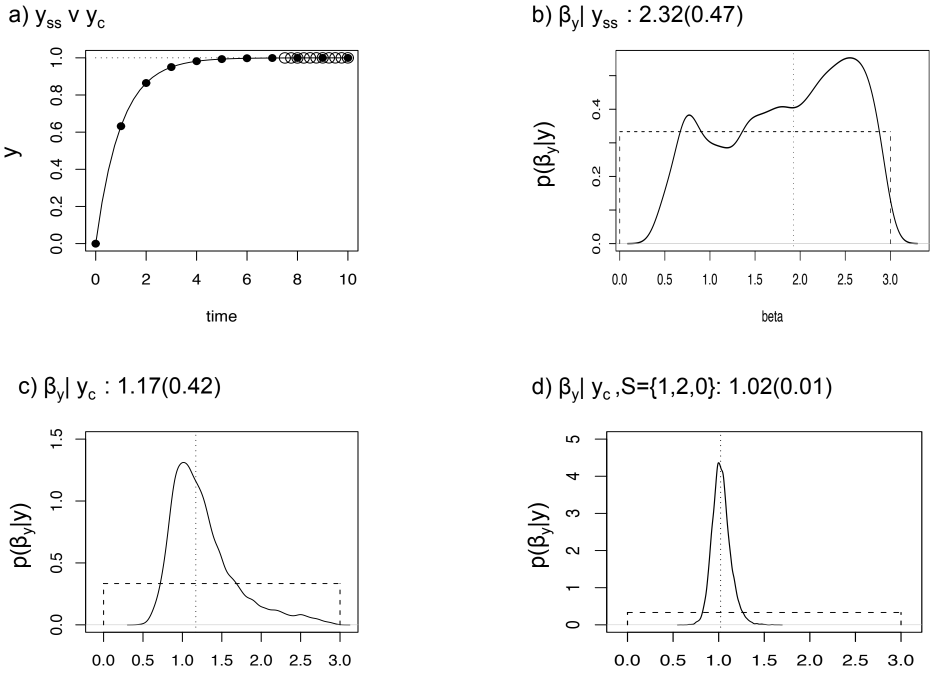

Figure S1: Selecting appropriate data intervals and signal function for parameter inference. In (a) datasets of the same size are sampled from the steady state zone (empty circles), or the complete time series (filled circles) for $S = 1$. Inference from the steady state data, y_{ss}, fails to converge and yields the posterior distribution shown in (b) for β_y (α_y behaves similarly). Inference from the complete dataset, y_c, is shown in (c). In (d), β_y is inferred with a richer signal function $S = \{1, 2, 0\}$. The same prior distributions, namely $\beta_y, \alpha_y \sim U[0, 3]$, were used in all cases (dashed boxes). The posterior median (dotted vertical line) and posterior standard deviation values are given in each figure.

Feedforward control models

Cartoon representations for the control models of the arabinose, flagella and galactose system are drawn in figure S3. Model reconstructions for the flagella dataset are shown in figure S4.

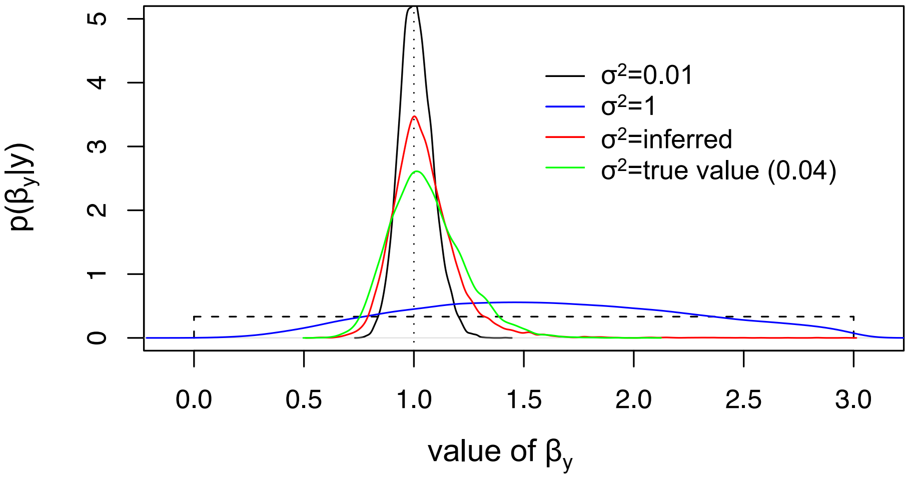

Figure S2: Error model treatment and accuracy of rate parameter estimates. Posterior densities for the parameter β_y under different noise specifications (see legend within plot). The prior on β_y is shown by a dashed box. In the case where the variance is estimated, an inverse Gamma prior with shape and scale parameters of 0.5 and 0.05 is used.

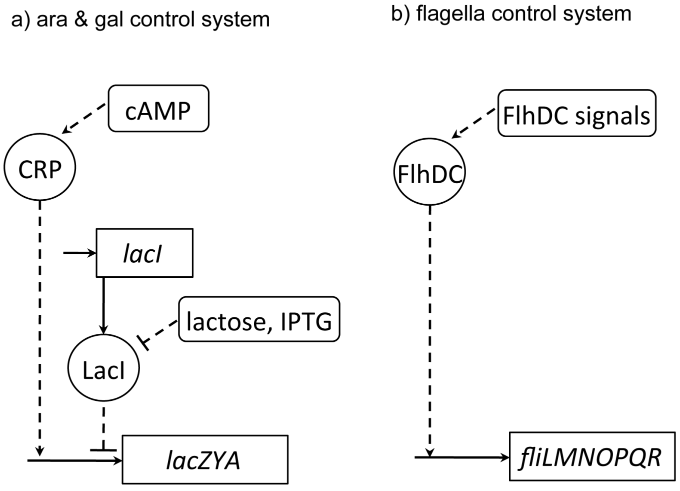

Figure S3: Control models for feed forward systems. The control system for the arabinose and galactose systems in *E.coli* is shown in (a). Note that in the presence of lactose or IPTG, both system branches are activating (coherent). The system is incoherent when the repressor LacI is not inhibited. The control system for the flagella network is shown in (b). Figures are adapted from Mangan *et al.* (2003) and Kalir *et al.* (2005).

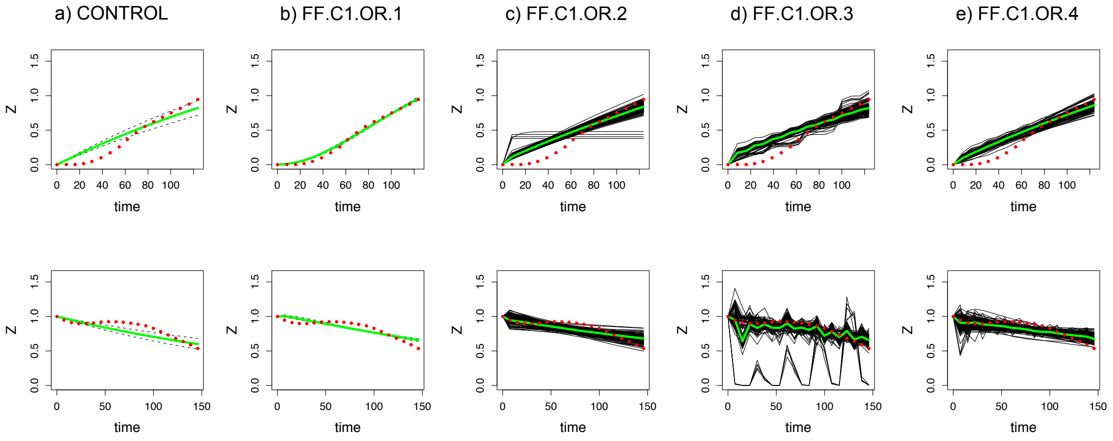

Figure S4: Reconstructing the flagella dataset. Model reconstruction for the flagella dataset (Mangan *et al.* 2006) using MCMC estimated models. These are the candidate models CONTROL, FF.C1.OR.1, FF.C1.OR.2, FF.C1.OR.3 and FF.C1.OR.4. The first row shows ON steps, while OFF steps are given in the second row.
